# Utilization Efficiency of Human Milk Oligosaccharides by Human-Associated *Akkermansia* Is Strain Dependent

**DOI:** 10.1128/AEM.01487-21

**Published:** 2022-01-11

**Authors:** Estefani Luna, Shanthi G. Parkar, Nina Kirmiz, Stephanie Hartel, Erik Hearn, Marziiah Hossine, Arinnae Kurdian, Claudia Mendoza, Katherine Orr, Loren Padilla, Katherine Ramirez, Priscilla Salcedo, Erik Serrano, Biswa Choudhury, Mousumi Paulchakrabarti, Craig T. Parker, Steven Huynh, Kerry Cooper, Gilberto E. Flores

**Affiliations:** a Department of Biology, California State University, Northridge, Northridge, California, USA; b GlycoAnalytics Core, UC San Diego, Health Sciences, La Jolla, California, USA; c Produce Safety and Microbiology Research Unit, Western Regional Research Center, Agricultural Research Service, U.S. Department of Agriculture, Albany, California, USA; d School of Animal and Comparative Biomedical Sciences, University of Arizona, Tucson, Arizona, USA; University of Manchester

**Keywords:** *Akkermansia muciniphila*, human milk oligosaccharides, fucosylated HMO, sialylated HMO, HMO utilization, *Akkermansia* phylogroups, glycoside hydrolase (GH), GH29, GH95

## Abstract

Akkermansia muciniphila is a mucin-degrading bacterium found in the human gut and is often associated with positive human health. However, despite being detected by as early as 1 month of age, little is known about the role of *Akkermansia* in the infant gut. Human milk oligosaccharides (HMOs) are abundant components of human milk and are structurally similar to the oligosaccharides that comprise mucin, the preferred growth substrate of human-associated *Akkermansia*. A limited subset of intestinal bacteria has been shown to grow well on HMOs and mucin. We therefore examined the ability of genomically diverse strains of *Akkermansia* to grow on HMOs. First, we screened 85 genomes representing the four known *Akkermansia* phylogroups to examine their metabolic potential to degrade HMOs. Furthermore, we examined the ability of representative isolates to grow on individual HMOs in a mucin background and analyzed the resulting metabolites. All *Akkermansia* genomes were equipped with an array of glycoside hydrolases associated with HMO deconstruction. Representative strains were all able to grow on HMOs with various efficiencies and growth yields. Strain CSUN-19, belonging to the AmIV phylogroup, grew to the highest level in the presence of fucosylated and sialylated HMOs. This activity may be partially related to the increased copy numbers and/or the enzyme activities of the α-fucosidases, α-sialidases, and β-galactosidases. This study examines the utilization of individual purified HMOs by *Akkermansia* strains representing all known phylogroups. Further studies are required to examine how HMO ingestion influences gut microbial ecology in infants harboring different *Akkermansia* phylogroups.

**IMPORTANCE** Human milk oligosaccharides (HMOs) are the third most abundant component of breast milk and provide several benefits to developing infants, including the recruitment of beneficial bacteria to the human gut. *Akkermansia* strains are largely considered beneficial bacteria and have been detected in colostrum, breast milk, and young infants. A. muciniphila Muc^T^, belonging to the AmI phylogroup, contributes to the HMO deconstruction capacity of the infant. Here, using phylogenomics, we examined the genomic capacities of four *Akkermansia* phylogroups to deconstruct HMOs. Indeed, each phylogroup contained differences in their genomic capacities to deconstruct HMOs, and representative strains of each phylogroup were able to grow using HMOs. These *Akkermansia*-HMO interactions potentially influence gut microbial ecology in early life, a critical time for the development of the gut microbiome and infant health.

## INTRODUCTION

Akkermansia muciniphila is a mucin-degrading specialist that colonizes the mucus layer of the human gastrointestinal tract (1). Paradoxically, *Akkermansia* also promotes mucus production by enhancing the differentiation of gut epithelial cells, thereby influencing mucosal homeostasis (2). Numerous positive associations have been observed between this bacterial lineage and human health. In adults, a decreased abundance of *Akkermansia* is associated with metabolic impairments (3), ulcerative colitis (4), and inflammatory bowel disease (5). In infants, a decrease in mucosal residents such as *Akkermansia* is associated with a compromised immune system and the development of atopic dermatitis (6).

The mechanisms by which A. muciniphila benefits human health appear to be directly linked to its ecological niche along the human gastrointestinal tract. Specifically, *A. muciniphila* bacteria colonize the oxic-anoxic interface of the mucus layers adjacent to host epithelial cells where they degrade host-produced mucins (7). Mucins are the main structural components of mucus and are composed of polypeptide chains rich in serine, threonine, and proline residues that are *O*-linked to a variety of oligosaccharides (8). These oligosaccharide side chains are comprised of *N*-acetylgalactosamine (GalNAc), *N*-acetylglucosamine (GlcNAc), and galactose and are capped with *N*-acetylneuraminic acid (Neu5Ac) (sialic acid), fucose, or sulfate. *Akkermansia* can utilize mucins as its sole carbon and nitrogen source, generating metabolites such as acetate, succinate, and propionate in the presence of vitamin B_12_ (9, 10). Co-occurring members of the gut microbiome convert some of the acetate produced to butyrate (11). Together, these organic acids fuel colonocytes and act as signaling molecules helping to maintain an overall anti-inflammatory tone in the gut (12). In addition to producing anti-inflammatory metabolites, *A. muciniphila* produces an extracellular surface protein, encoded by Amuc_1100, that interacts directly with Toll-like receptors on host epithelial cells (13, 14). This interaction results in the production of specific anti-inflammatory cytokines, including interleukin-10 (IL-10), which leads to an improvement in overall gut barrier function (13).

Building upon previous work by Guo and colleagues (15), we recently performed a comparative genomic analysis of 75 *Akkermansia* genomes to define the genomic and functional landscape of this lineage. This analysis identified at least four distinct phylogroups, AmI to AmIV, with *A. muciniphila* Muc^T^ belonging to the AmI phylogroup. Additionally, this work showed that the *Akkermansia* phylogroups had differing functional potentials, including *de novo* biosynthesis of vitamin B_12_ by members of the AmII and AmIII phylogroups (10).

Continuing to explore the genomic and metabolic diversity of human-associated *Akkermansia*, we next wanted to determine if host-produced glycans, other than those in mucin, could support the growth of various *Akkermansia* phylogroups. Because of the compositional and structural similarities between the oligosaccharides found in mucin and human milk, we focused on human milk oligosaccharides (HMOs) (8, 16, 17). Human milk contains 5 to 15 g/L HMOs, of which 50 to 80% are fucosylated and 10 to 20% are sialylated (16). Although HMOs are present in milk as a pool of over 200 diverse structures, they are composed of only five monosaccharides: glucose, galactose, fucose, GlcNAc, and sialic acid (16). These oligosaccharides contain a lactose core at the reducing end that is extended with building block monosaccharides via glycosidic linkages. In human milk, fucose can be attached via α1-2, α1-3, and α1-4 linkages, and sialic acid can be attached via α2-3 and α2-6 linkages. Simple, abundant, and routinely studied HMO structures include lacto-*N*-tetraose (LNT), lacto-*N*-neotetraose (LNnT), 2′-fucosyllactose (2′-FL), 3-fucosyllactose (3-FL), 6′-sialyllactose (6′-SL), and 3′-sialyllactose (3′-SL) (18).

The oligosaccharides found in human milk are not digestible by the developing infant and reach the intestine intact (19). Once there, HMOs have a variety of functions, including providing protection from pathogens, playing a role in the modulation of gut epithelial cells, and enriching for a beneficial microbiota (20–22). Several studies have screened HMO consumption by various intestinal commensals and have identified a limited group of bacteria, primarily *Bifidobacterium* and select *Bacteroides* species, with this ability (23–25). One *Akkermansia* strain, belonging to phylogroup AmI (i.e., *A. muciniphila* Muc^T^), has recently been shown to grow on human milk and select HMOs using a repertoire of glycoside hydrolase (GH) enzymes (26). In this study, we expand our understanding of this HMO-degrading capacity of human-associated *Akkermansia* beyond the one phylogroup. We hypothesized that *Akkermansia* strains from different phylogroups will differ in their abilities to metabolize HMOs, and these differences are related to their genomic composition. To investigate the ability of *Akkermansia* to grow on select HMOs, we first took a comparative genomics approach focusing on the presence and abundance of genes coding for glycoside hydrolase enzymes known to be involved in HMO catabolism. We then performed comparative growth experiments and demonstrated the robust growth of one representative strain from each of the four phylogroups in a basal medium supplemented with five individual pure HMOs, in a background of mucin, thus simulating the carbon sources available in the infant gut environment. These findings expand the known metabolic capabilities of human-associated *Akkermansia* and point to further functional differences among the genomically distinct phylogroups.

## RESULTS

### Newly isolated *Akkermansia* strains differ in their phylogenomic characteristics.

**FIG 1:**
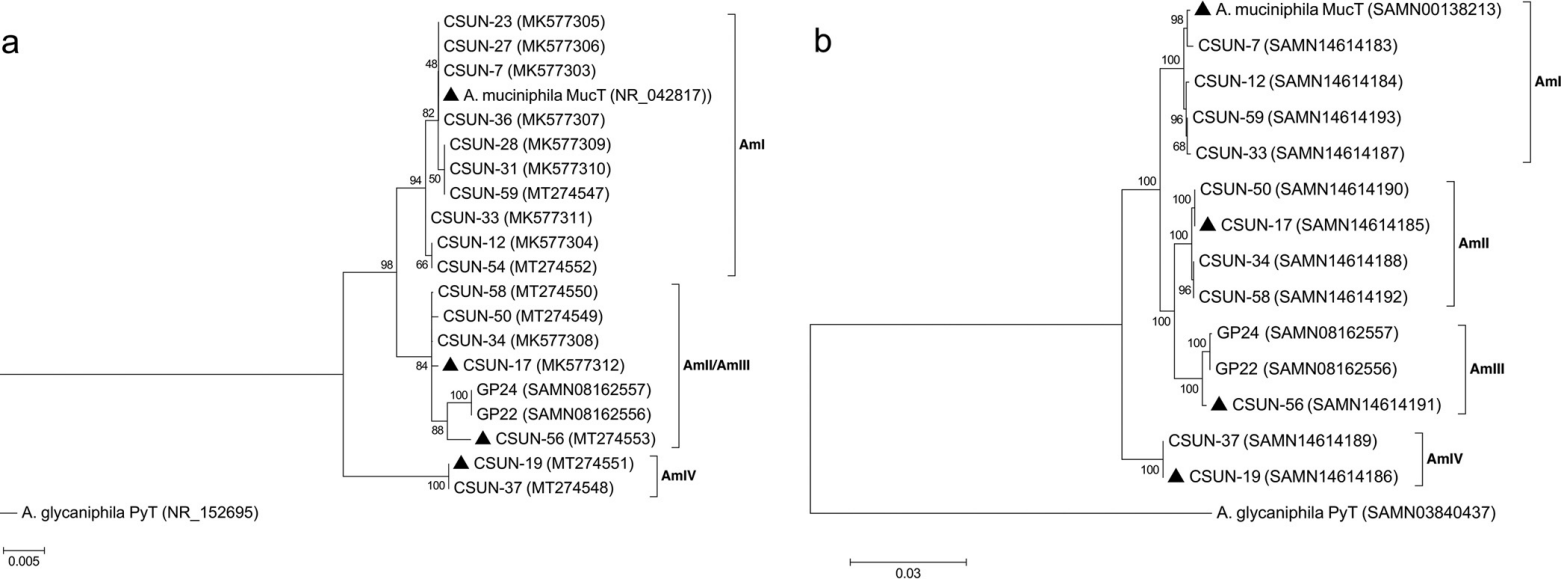
Phylogenetic relationship of *Akkermansia* isolates based on nearly full-length 16S rRNA gene sequences (a) and concatenation of 49 ribosomal protein-coding genes obtained from draft genomes (b). Both trees are rooted using the only other named species of the genus, Akkermansia glycaniphila Py^T^. Isolates with triangles were used in HMO growth experiments. GP22 and GP24 in the AmIII phylogroup are from Guo and colleagues (15) and are included because only one AmIII representative is available in our culture collection. Both trees were generated in MEGA7 (68) using the maximum likelihood method, and numbers at the nodes indicate bootstrap values for 100 replicates. The tree in panel a was generated considering only unambiguously aligned nucleotide positions (*n* = 1,305). For panel b, a total of 7,327 amino acid positions across 49 protein-coding genes were used. Both trees are drawn to scale, with branch lengths measured in the number of substitutions per site. GenBank accession numbers are in parentheses.

In total, 17 human-associated *Akkermansia* strains were isolated from healthy adults, 10 from males and 7 from females (see Table S2 in the supplemental material). Phylogenetic analyses of the nearly complete 16S rRNA gene sequences from each isolate revealed three well-supported clades, with the AmIII phylogroup nested within the AmII phylogroup (Fig. 1a). At least one isolate was obtained from the four known human-associated phylogroups (10). Ten of the 17 isolates treed within the AmI phylogroup, followed by 4 in AmII, 2 in AmIV, and 1 in AmIII.

Using the 16S rRNA tree as a guide, we selected 11 of the isolates spanning each phylogroup for genomic sequencing. The characteristics of these draft genomes are presented in Table 1. Of the new isolates, draft genome sizes ranged from 2.86 Mb (CSUN-56 [AmIII]) to 3.15 Mb (CSUN-19 [AmIV]), with 2,658 to 3,111 coding sequences (CDs), respectively, compared with a 2.67-Mb genome size and 2,576 CDs in *A. muciniphila* Muc^T^. Across phylogroups, approximately 52% of CDs could be assigned a function, on average. The resolution of the AmIII phylogroup was improved by phylogenomic analysis that included 49 protein-coding genes (Fig. 1b).

**TABLE 1:** Genomic properties of 11 human-associated *Akkermansia* isolates^*a*^

Strain (phylogroup)	No. of contigs	GC content (%)	Genome length (bp)	No. of CDs	No. of tRNAs	No. of rRNAs	No. of hypothetical proteins	No. of proteins with assignments	No. of EC no. assignments	No. of GO assignments	No. of KEGG pathways
*A. muciniphila* Muc^T^ (AmI)	1	55.8	2,664,102	2,576	53	3	1,072	1,504	620	529	459
*Akkermansia* CSUN-7 (AmI)	52	55.1	2,875,736	2,880	50	3	1,377	1,503	623	530	464
*Akkermansia* CSUN-12 (AmI)	56	55.3	2,810,203	2,823	50	3	1,307	1,516	632	538	465
*Akkermansia* CSUN-33 (AmI)	49	55.3	2,833,117	2,853	50	3	1,307	1,546	633	541	469
*Akkermansia* CSUN-59 (AmI)	65	55.2	2,942,175	3,010	50	3	1,472	1,538	628	535	466
*Akkermansia* CSUN-17 (AmII)	29	58.2	2,999,178	2,856	49	3	1,354	1,502	641	542	474
*Akkermansia* CSUN-34 (AmII)	87	57.8	3,024,116	2,949	47	3	1,451	1,498	640	538	473
*Akkermansia* CSUN-50 (AmII)	23	58.2	3,005,559	2,842	49	3	1,365	1,477	632	533	470
*Akkermansia* CSUN-58 (AmII)	71	57.8	3,087,515	2,988	49	3	1,489	1,499	637	535	472
*Akkermansia* CSUN-56 (AmIII)	48	58.5	2,860,685	2,658	48	3	1,246	1,412	612	518	462
*Akkermansia* CSUN-19 (AmIV)	89	56.6	3,149,202	3,111	49	3	1,656	1,455	628	535	472
*Akkermansia* CSUN-37 (AmIV)	72	56.7	3,142,630	3,077	49	3	1,631	1,446	624	531	469

a For comparison, the fasta sequence of Akkermansia muciniphila Muc^T^ was downloaded from GenBank (accession number CP001071.1) and analyzed identically to the new isolates. CDs, coding sequences; EC, Enzyme Classification; GO, Gene Ontology; KEGG, Kyoto Encyclopedia of Genes and Genomes.

**FIG 2:**
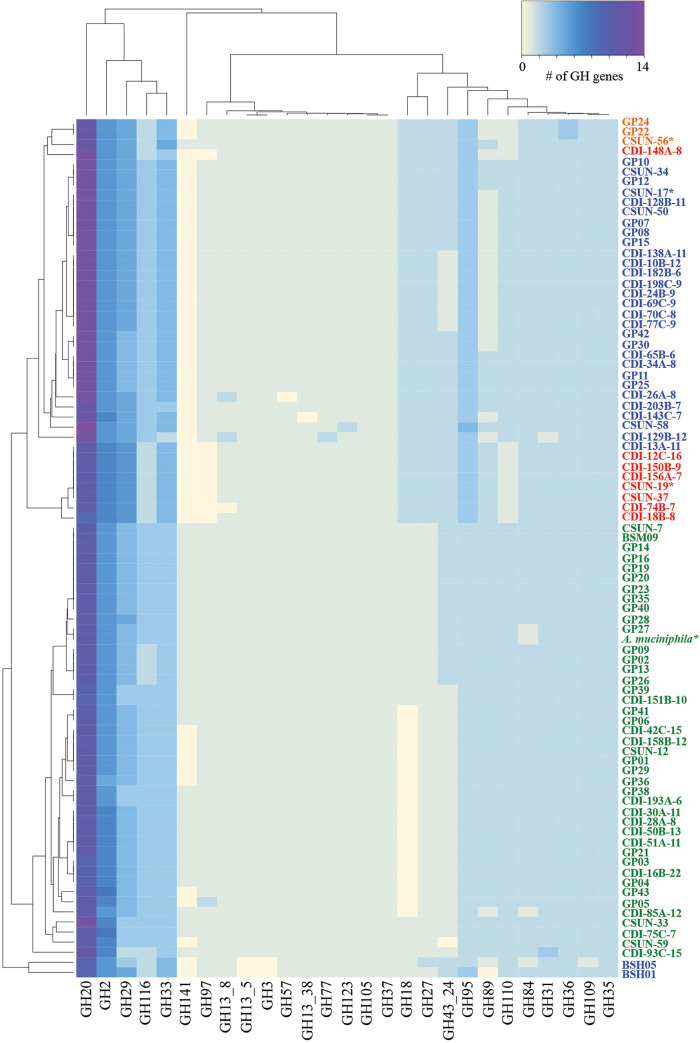
Human-associated *Akkermansia* strains possess different complements of glycoside hydrolase (GH) genes potentially impacting their carbohydrate-degrading capabilities. The heat map shows the counts of different GH families present in the draft genomes of 85 total *Akkermansia* genomes. Genomes labeled with “CSUN” prefixes are isolates from this work, while the “CDI” genomes are from metagenome-assembled genomes (10), and the “GP” or “BSM” genomes are from isolates from Guo and colleagues (15). Each genome is colored by phylogroup affiliation. Green, AmI; blue, AmII; orange, AmIII; red, AmIV. Only three genomes (CDI-148A-8, BSH05, and BSH01) tree outside their phylogroup affiliation based on the GH content. Genomes with asterisks were used in the HMO growth experiments.

To investigate the carbohydrate-degrading potential of the *Akkermansia* strains, 85 genomes, including the 11 isolates from this study, were annotated against the CAZy database (27) using dbCAN (28, 29). We first took a global look at all annotated GH families and found significantly fewer GH annotations in genomes from the AmI phylogroup than in those from the other phylogroups (χ^2^ = 55.128; *P* < 0.0001 [by a Kruskal-Wallis test]) (Fig. S2). Furthermore, we identified consistent similarities and differences in the complements of GH annotations within and between each phylogroup (Fig. 2). With a few minor exceptions, these similarities and differences in GH counts resulted in the clustering of genomes into their respective phylogroups as evidenced by the dendrogram along the *y* axis in Fig. 2.

Next, since we were interested in the ability of *Akkermansia* to degrade HMOs, we focused on HMO-associated GH families previously identified in other organisms (26, 30–33). With this approach, we identified differences in the copy numbers of several GH families that are associated with the degradation of HMO glycans: α-fucosidases, α-sialidases, β-galactosidases, and *N*-acetyl β-hexosaminidases (Table 2). Most of these genes were also found to possess a signal peptide (Data Set S1), which is indicative of encoding extracellular enzymes (34, 35). Of note was the high number of GH20 genes compared with any other GH gene in all the genomes. The numbers of putative α-fucosidases (GH29, GH95, and GH141) and *N*-acetyl β-hexosaminidases (GH18, GH20, GH84, and GH109) also varied across phylogroups (lowest for AmI, including the strain tested here, *A. muciniphila* Muc^T^). Of the four strains investigated for HMO catabolic capacity in this study, the CSUN-19 (AmIV phylogroup) and CSUN-56 (AmIII) strains showed 9 fucosidase annotations, compared with 8 for CSUN-17 (AmII) and 7 for *A. muciniphila* Muc^T^ (AmI) (Table 2).

**TABLE 2:** Copy numbers of several human milk oligosaccharide-associated glycoside hydrolase families in representative strains from the different *Akkermansia* phylogroups

Glycoside hydrolase family	Enzyme activity(ies)	Copy no. (avg copy no.)			
		*A. muciniphila* Muc^T^ (AmI)	CSUN-17 (AmII)	CSUN-56 (AmIII)	CSUN-19 (AmIV)
GH2	β-Galactosidase (or similar)	6 (6.3)	6 (6)	6 (6)	7 (6.9)
GH16	β-Galactosidase (or similar)	3 (2.9)	3 (2.9)	2 (2)	2 (2)
GH18	Chitinase; endo-β-*N*-acetylglucosaminidase (or similar)	1 (0.5)	2 (1.9)	2 (2)	2 (2)
GH20	β-Hexosaminidase; lacto-*N*-biosidase; β-*N*-acetylglucosaminidase	11 (11)	13 (12.8)	12 (12)	11 (11)
GH29	α-l-Fucosidase	4 (3.8)	5 (4.7)	5 (5)	6 (5.9)
GH33	Sialidase (or similar)	3 (3)	4 (3.9)	5 (4.3)	4 (3.9)
GH35	β-Galactosidase; exo-β-glucosaminidase	2 (2)	2 (2)	2 (2)	2 (2)
GH84	*N*-Acetyl β-glucosaminidase; hyaluronidase	1 (1.9)	2 (2)	2 (2)	2 (2)
GH95	α-l-Fucosidase; α-l-galactosidase	2 (2)	3 (3)	3 (3)	3 (3)
GH109	α-*N*-Acetylgalactosaminidase; β-*N*-acetylhexosaminidase	2 (2)	2 (2)	2 (2)	2 (2)
GH141	α-l-Fucosidase; xylanase	1 (0.8)	0 (0)	1 (0.3)	0 (0)

### The four *Akkermansia* strains show strain-dependent growth and utilization of HMOs.

**FIG 3:**
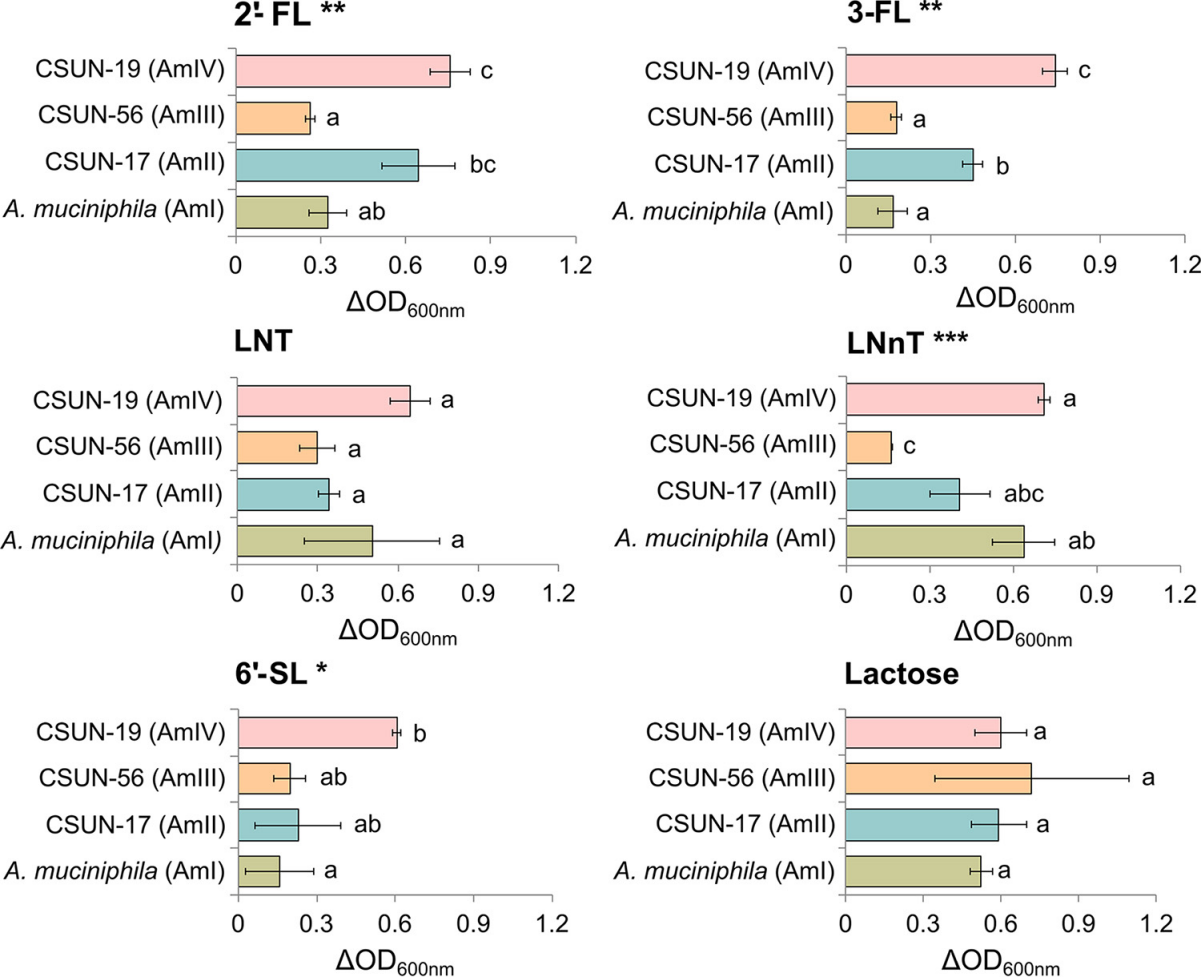
A representative strain from each of the four *Akkermansia* phylogroups was incubated in mucin-containing medium alone or supplemented with 20 mM individual human milk oligosaccharides or lactose. The experiment was conducted in triplicate and repeated at least two times. The difference in the OD_600_ from the growth in mucin-containing medium alone was used to plot the bacterial growth for each strain. Values are expressed as averages ± standard deviations. ANOVAs reveal significant effects (**, *P* < 0.01; *, *P* < 0.05) with the substrates 2′-fucosyllactose (2′-FL), 3-fucosyllactose (3-FL), lacto-*N*-neotetraose (LNnT), and 6′-sialyllactose (6’-SL) but not with lacto-*N*-tetraose (LNT) and lactose. Pairwise comparisons within each substrate using Tukey’s honestly significant difference test reveal significant differences between the phylogroups (*P* < 0.05); means showing letters in common are not significantly different.

One representative of each of the four phylogroups was tested for its ability to grow on HMO in the presence of mucin. After 48 h of incubation, all strains tested grew to higher optical densities (ODs) in HMO (or lactose)-supplemented mucin medium than in medium lacking HMOs (Fig. 3). Growth yields varied across strains on media with 2′-FL, 3-FL, LNnT, and 6′-SL but not LNT or lactose (*P* < 0.05 by analysis of variance [ANOVA]). *Post hoc* comparisons revealed that strain CSUN-19, representing the AmIV phylogroup, showed the greatest growth in comparison to the other strains, with significant increases compared with *A. muciniphila* Muc^T^ in 2′-FL, 3-FL, and 6′-SL and with CSUN-56 in 2′-FL, 3-FL, and LNnT (Fig. 3).

**FIG 4:**
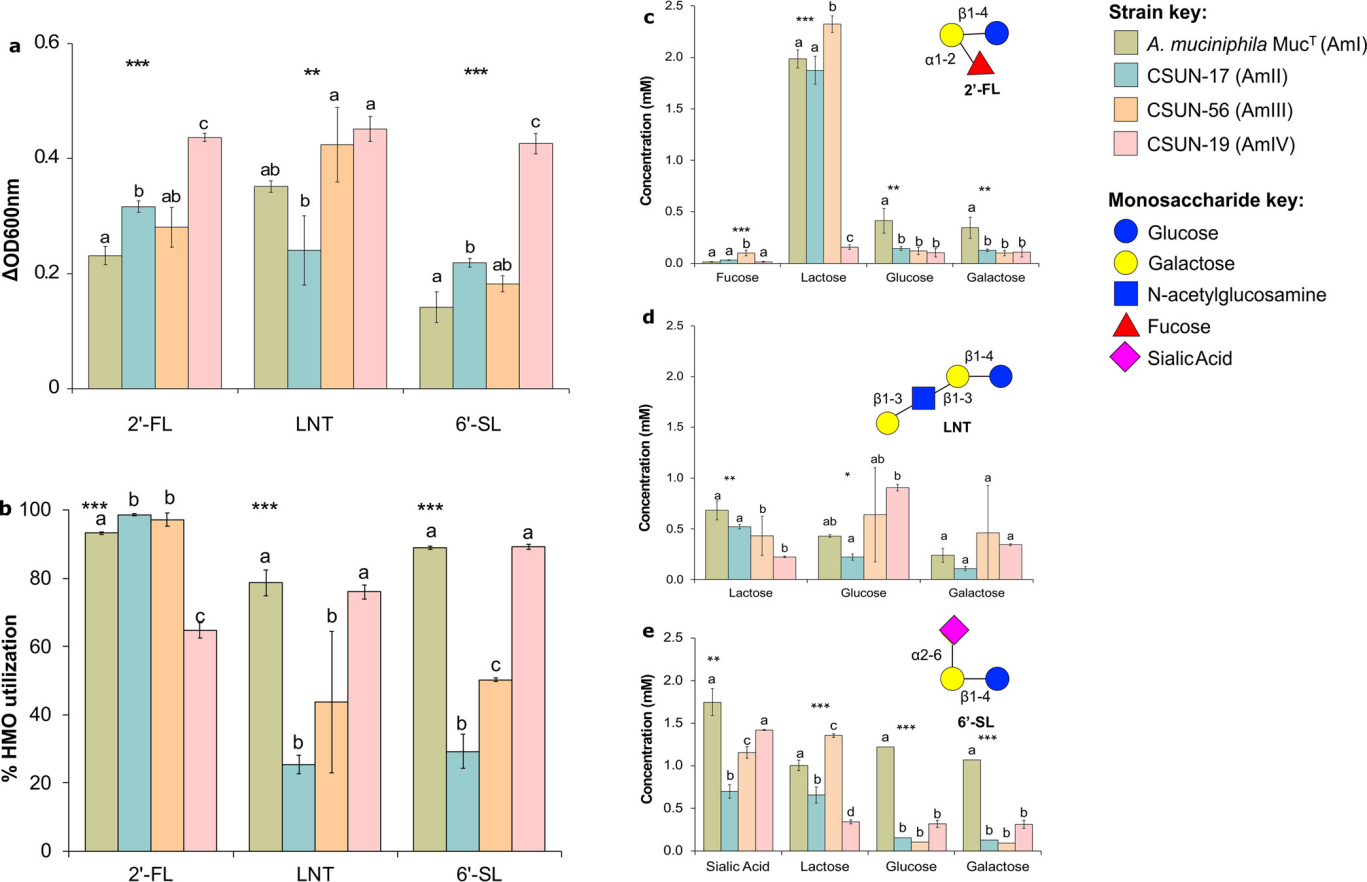
A representative strain from each of the four *Akkermansia* phylogroups was incubated in mucin-containing medium alone or supplemented with 4 mM individual human milk oligosaccharides (HMOs) or lactose. The experiment was conducted in triplicate and repeated three times. (a) The difference in growth in HMO-supplemented medium from the growth in mucin-containing medium alone was used to plot the bacterial growth for each strain. (b) The concentrations of the original substrate analyzed were used to calculate the percentage of the HMO utilized. (c to e) Concentrations of the metabolites obtained after the deconstruction of 2′-fucosyllactose (2′-FL) (c), lacto-*N*-tetraose (LNT) (d), and 6′-sialyllactose (6′-SL) (e) expressed as averages ± standard deviations. Statistical analysis revealed significant effects between the substrates (a and b) and strains (b to d) (***, *P* < 0.001; **, *P* < 0.01; *, *P* < 0.05). Pairwise comparisons using Tukey’s honestly significant difference test were also performed, with a *P* value of <0.05, and means showing letters in common are not significantly different.

To confirm HMO utilization, we measured the concentrations of HMOs (2′-FL, LNT, and 6′-SL) and their sugar constituents (except GlcNAc for LNT) before and after 48 h of incubation (Fig. 4a and b). In addition to the differences in growth yields, the differences in the percentages of HMO utilized also varied across strains (*P* < 0.05). For 2′-FL, strains representing the AmI, AmII, and AmIII phylogroups utilized >93% of the available HMO, while CSUN-19 (AmIV) utilized just over 64% despite having the highest growth yield as measured by the change in the OD at 600 nm (OD_600_). Nearly all of the fucose liberated from 2′-FL was removed from the medium within 48 h by all the strains, while the lactose backbone accumulated in the culture medium of all strains except CSUN-19 (AmIV) (Fig. 4c). The degradation of LNT ranged from 25.4 to 78.6% across the tested strains, with CSUN-17 (AmII) utilizing the least and *A. muciniphila* Muc^T^ (AmI) utilizing the most. In contrast to growth on 2′-FL, most of the lactose from LNT was consumed across strains (Fig. 4d). Similar to LNT, there was a wide range of 6′-SL utilizations across strains (*P* < 0.001), ranging from 29.3% (CSUN-17 [AmII]) to 89.2% (CSUN-19 [AmIV]). In the case of 6′-SL, CSUN-19 showed the greatest growth, while *A. muciniphila* Muc^T^ showed the least growth, and yet the percentage of the substrate utilized (80%) showed no significant difference and was significantly higher than the ∼50% and ∼30% utilizations seen with CSUN-56 and CSUN-17, respectively. In all strains, sialic acid accumulated in the culture media and was not consumed when liberated from 6′-SL (Fig. 4e).

## DISCUSSION

*Akkermansia* strains are largely considered beneficial members of the human gut microbiome and are currently of significant interest for their therapeutic potential (36). Until recently, however, all research involving these promising bacteria focused on a single species, *A. muciniphila* Muc^T^, belonging to the AmI phylogroup. Here, we continue to build upon recent work by us and others describing the genomic and functional diversity within this lineage (10, 37). Specifically, we show that genomically diverse strains possess different complements of glycoside hydrolase (GH) genes that encode enzymes catalyzing the deconstruction of HMOs into constituent mono- and disaccharides. Furthermore, this study demonstrates that four different *Akkermansia* strains representing the four known phylogroups can deconstruct all the major types of HMOs, with this biological activity varying across strains. These differences in genomic and functional traits of human-associated *Akkermansia* strains, along with the diversity in the substrates that are presented to the gut bacteria in the form of breast milk or supplemented infant milk formula, potentially impact how and when *Akkermansia* strains colonize the human gastrointestinal tract. Thus, since HMO-*Akkermansia* interactions are strain specific, the corresponding pattern of early colonization with human-associated *Akkermansia* and the ensuing competitive advantage could also be strain specific in infants ingesting HMOs. *Akkermansia* bacteria are key contributors to the infants’ glycan-metabolizing capacity by as early as 4 months of age (33) and may therefore play a critical role in establishing a foundation of metabolic fitness in the naive microbiome. Taken together, these findings expand the known metabolic niche and interaction network of *Akkermansia* in the human gut early in life.

Bacterial growth studies have demonstrated that relatively few gut bacteria grow well on HMOs, the exceptions being bifidobacteria and select *Bacteroides* strains, both of which are dominant members of the infant gut (24, 25). Both bifidobacteria and *Bacteroides* employ an array of glycoside hydrolases, including fucosidases (GH29 and GH95), sialidases (GH33), galactosidases (GH2 and GH16), lacto-*N*-biosidases (GH20), and hexosaminidases (GH20), to deconstruct HMO linkages (38–44). Our phylogenomic characterization of the *Akkermansia* genomes shows that the various strains from the four *Akkermansia* phylogroups possess a wealth of these same gene annotations, albeit in differing abundances, that could be used for the deconstruction of either HMO or mucin. Genomes of the AmII, AmIII, and AmIV phylogroups were found to contain a higher number of genes encoding GH18, GH29, and GH95 than the genomes of the AmI phylogroup. This is consistent with findings by Becken and colleagues for AmII and AmIV (AmIII was not studied there) (37). They also found that the AmI phylogroup may be phylogenetically divided into two subclades, AmIa and AmIb, with *A. muciniphila* Muc^T^ belonging to subclade AmIa. Using this framework, they found differences in the complements of GH genes between AmIa and AmIb. Specifically, the AmIb subclade had few GH29-encoding genes and no GH18 genes compared to the AmIa subclade. This explains the lower average number of GH18 and GH29 genes in the AmI phylogroup that we observed and suggests that differences in carbohydrate catabolism likely exist within *Akkermansia* phylogroups.

Bifidobacteria employ two major strategies to hydrolyze HMOs (31, 42). Infant-associated Bifidobacterium infantis, Bifidobacterium breve, and Bifidobacterium longum primarily consume HMOs by employing intracellular glycoside hydrolases to deconstruct the HMO structures (41, 42, 45–47). Using an alternative strategy, Bifidobacterium bifidum extracellularly processes HMO via an array of membrane-associated glycoside hydrolases (31, 48). *Bacteroides* spp. harbor polysaccharide utilization loci (PULs) that encode a diverse array of glycosidases capable of breaking down host-produced and plant-derived polysaccharides (44, 49). *Bacteroides* bacteria are hypothesized to bind HMOs on the cell surface followed by the hydrolysis of the HMOs and the import of the resultant oligosaccharides for further breakdown. They co-opt their mucin utilization PULs to deconstruct and utilize HMOs with various efficiencies depending on the strain. Bacteroides fragilis is the most efficient in preferring HMOs with a high degree of polymerization and nonfucosylated HMOs over fucosylated HMOs (24) and even utilizes the sialic acid generated after the deconstruction of sialylated HMOs (44). *Akkermansia* does not have the typical PUL genomic organization seen in *Bacteroides* but it appears to harness extracellular GHs either in the periplasmic space or outside the cell altogether to cleave monosaccharides or disaccharides from mucin or HMOs (9, 26). In agreement, the majority of our GH annotations included signal peptide sequences indicative of export outside the cytoplasmic membrane. The extracellular cleavage of HMO (and mucin) results in the liberation of monosaccharides and disaccharides, which enables cross-feeding by other members of the gut microbiome (11). In the context of the infant gut, this cross-feeding could help facilitate colonization by new members of the gut community that are encountered as infants grow and consume new foods, aiding in the maturation of the gut microbiome in the early years of life (50).

In addition to cross-feeding on sugars liberated from host substrates, members of the gut microbiome feed off fermentation waste products produced by *Akkermansia* (26, 51, 52). In the case of fucosylated substrates such as 2′-FL, a distinct metabolite of fucose fermentation is 1,2-propanediol (26). Several bacteria, including both beneficial (*Lactobacillus* spp. and Eubacterium hallii) and pathogenic (Salmonella) bacteria, can grow on 1,2-propanediol in a vitamin B_12_-dependent manner (51, 52). Given our recent work showing that the AmII and AmIII phylogroups synthesize vitamin B_12_ (10), these findings indicate the possibility of *Akkermansia*-driven syntrophic interactions that are likely phylogroup specific. This is particularly relevant as the gut microbiome of exclusively breastfed infants has a decreased capacity for the *de novo* synthesis of vitamin B_12_ compared with formula-fed infants (53). Therefore, an understanding of which *Akkermansia* strain is already present in a host may influence the outcome of any microbiome or dietary intervention.

Several studies have detected *Akkermansia* in the stool of infants as early as 1 month after birth, in most 1-year-olds (54), and even in human colostrum and milk (55, 56), demonstrating that it colonizes the gut early in life and providing a possible route of inoculation. Two separate studies found direct associations between the abundances of *Akkermansia* and fucosylated HMO in human milk, suggesting that fucosylated HMO may help enrich for *Akkermansia* in the gut of the infant (57, 58). Here, we show that fucosylated HMOs support robust growth across all strains of *Akkermansia*. However, growth varied by strain, suggesting potential differences in growth and metabolic efficiencies across strains. When grown on 2′-FL, the liberated fucose was rapidly depleted from the culture medium, while the lactose component accumulated in the culture medium (except for CSUN-19), suggesting a general preference for fucose over lactose in *Akkermansia*. Cleavage of fucose from HMOs (and mucin) is mediated by fucosidases belonging to the GH29 and GH95 families (31, 38, 49), both of which were found in all four *Akkermansia* strains. GH141, a putative fucosidase or xylanase, was also observed in some of our AmI and AmIII genomes in this study. Kostopoulos et al. recently demonstrated that a GH29 gene product (encoded by Amuc_0010) in *A. muciniphila* Muc^T^ had relatively poor catalytic activity against 2′-FL, suggesting that 2′-FL was not the preferred substrate for this enzyme (26). It should be cautioned that *A. muciniphila* Muc^T^ belongs to subclade AmIa within the AmI phylogroup (37), hence the HMO utilization characteristics of this strain may not represent the capabilities of the whole AmI phylogroup. Overall, *A. muciniphila* Muc^T^ has four GH29 gene annotations, two of GH95, and one of GH141, and all these GH families could potentially encode enzymes that are involved in the degradation of fucosylated HMOs containing the α1-2 linkage. The numbers of these same GH families also varied across phylogroups, potentially leading to the differences in growth efficiencies that we observed. Given the prominent role of fucosylated HMOs in modulating the microbiome and enhancing health, and given that the concentrations of 2′-FL along with lacto-*N*-fucopentaose were highest during early lactation (59), the diversity of fucosidases available in each strain makes *Akkermansia* a potential candidate for further investigation in the field of infant-associated probiotics.

Sialyl oligosaccharides are associated with many benefits to neonates and infants (60, 61). For example, Charbonneau and colleagues demonstrated that the concentration of sialylated HMOs in breast milk correlated with growth in healthy Malawian infants (61). Furthermore, gnotobiotic mammals receiving fecal microbiota from infants with stunted growth and supplementation with sialylated bovine milk oligosaccharides showed improved growth (measured as weight gain and bone mass), with their gut microbiota developing metabolic fitness evidenced by an increase in genes related to energy metabolism (61). Sialic acid is an essential component of brain gangliosides and plays important roles in neuronal development, memory formation, and cognition (60). Three weeks of dietary supplementation with 3′-SL or 6′-SL administered to day-old piglets increased the ganglioside-bound sialic acid in the brains of the piglets, thus providing essential nutrients for brain growth and neurodevelopment (62). With regard to *Akkermansia* and sialylated HMOs, all four *Akkermansia* strains showed enhanced growth on 6′-SL and were able to deconstruct this sialylated oligosaccharide, but the growth yield and the percentage of the substrate degraded varied significantly across strains. These differences in yield and degradation did not align with the sialidase (GH33) gene copy number. For example, strain CSUN-56 representing AmIII has 5 sialidase annotations and exhibited relatively poor growth, with little degradation of 6′-SL. This incongruence between the bacterial gene number of a GH metabolizing a substrate and the physiological response to that substrate indicates the need to examine the transcription of the GHs and the enzyme kinetics of the associated GHs involved in the complete deconstruction of substrates and their transport into the cell. However, the accumulation of sialic acid in spent medium after growth on 6′-SL in all strains agrees with previous reports of *Akkermansia* lacking the *nan* operon for the import and consumption of sialic acid (26). The sialic acid released from the nonreducing end of the sugars enables access to the remaining oligosaccharides while also potentially encouraging the outgrowth of sialic acid-metabolizing, abundantly present commensal species such as B. fragilis, Faecalibacterium prausnitzii, Ruminococcus gnavus, and members of the *Lactobacillus* and *Bifidobacterium* genera (17, 63, 64). Several species of *Enterobacteriaceae* such as Escherichia coli and Salmonella enterica also thrive in a sialic acid-rich gut environment, with their fitness and virulence being directly proportional to their ability to metabolize sialic acid (63). Interestingly, though, studies in piglets demonstrated that supplementation with 6′-SL enhanced colonic bacteria such as Collinsella aerofaciens, *Ruminococcus*, *Faecalibacterium*, and *Prevotella* spp. while suppressing *Enterobacteriaceae*, *Enterococcaceae*, *Lachnospiraceae*, and *Lactobacillales* (62). Given the vulnerability of the infant population and the immaturity of the gut microbiome in early life, identifying the metabolic fate of sialic acid and the interaction between *Akkermansia* and sialic acid-metabolizing commensals and potential pathogens warrants further investigation.

*Akkermansia* strains are adapted to robust growth on mucin due to their habitation in the gut epithelial mucosa (65). Furthermore, HMOs, which are resistant to host digestive enzymes, are presented to the colonic microbiota in a mucin-rich background of the infant gut (66). We therefore included mucin in our HMO utilization experiments, and growth in the non-HMO mucin control served as a reference point to quantify HMO utilization. However, it is recognized that *Akkermansia* can grow in a mucin-deficient medium supplemented with GlcNAc, threonine, and tryptone (9). GlcNAc is a requirement for growth as *Akkermansia* does not express the enzyme required for the conversion of fructose-6-phosphate to glucosamine-6-phosphate, an essential component of the cell wall peptidoglycan (65). GlcNAc was thus added to the basal growth medium by Kostopoulos and colleagues while investigating HMO utilization by Akkermansia muciniphila Muc^T^ (26). We speculate that *Akkermansia* may grow exclusively using GlcNAc-containing HMOs such as LNT or LNnT, provided that the amino acid sources are added to the growth medium. However, since our current technique precluded analysis of GlcNAc, further growth experiments and chemical analyses are required to confirm this prediction.

In conclusion, human-associated *Akkermansia* strains can utilize a variety of host-derived HMOs for growth *in vitro* in a strain-dependent manner. This implies that the prebiotic effects of HMOs will depend on the resident strain of *Akkermansia* present in an individual. When grown on HMO, *Akkermansia* liberates sugars and produces fermentation products that can fuel other members of the gut microbiome. Together, these findings lay the foundation for future work examining the molecular mechanisms of HMO deconstruction by diverse strains of *Akkermansia* and how these activities influence interactions with the human host and other members of the gut microbiome in a strain-dependent manner.

## MATERIALS AND METHODS

### Recruitment and sampling.

Fecal samples used for *Akkermansia* isolations were obtained from 17 consenting healthy adults as previously described by Kirmiz et al. (10) under protocol number 1516-146, with approval from the Institutional Review Board at California State University, Northridge. Samples were refrigerated (4°C) and inoculated into culture medium (see below) within 24 h of collection.

### Bacterial isolation and identification.

*Akkermansia* isolation and identification were conducted as previously described (10). Briefly, 5 mL of anaerobic basal mucin medium (BMM) containing 0.5% (vol/vol) mucin (see Table S1 in the supplemental material) was inoculated with fecal swabs in serum tubes, and a 10-fold serial dilution of up to 10^−6^ or 10^−7^ was performed for each sample. Cultures were incubated at 37°C for up to 5 days, and those with oval cells in pairs were further diluted in broth medium and/or transferred to BMM agar until purity could be verified microscopically using a Zeiss Axioskop instrument or as single colonies on BMM agar. For identification, genomic DNA was extracted using the DNeasy UltraClean microbial isolation kit (Qiagen Inc., MD, USA), and the nearly full-length 16S rRNA gene was amplified using primers 8F (5′-AGAGTTTGATCCTGGCTCAG-3′) and 1492R (5′-TACGGTTACCTTGTTACGA-3′) with the GoTaq Hot Start colorless master mix (Promega Corp., Madison, WI, USA). PCR was performed using an Eppendorf Vapo Protect Mastercycler Pro S 6325 system (Hamburg, Germany) and included an activation/denaturation step at 95°C for 3 min; 30 cycles of 95°C for 45 s, 45°C for 1 min, and 72°C for 1 min 45 s; and a final extension step at 72°C for 7 min, followed by a hold at 4°C. PCR products were purified (QIAquick PCR purification kit; Qiagen Inc.) and sequenced using either the 8F or 1492R primer on an ABI Prism 3730 DNA sequencer (Laragen Sequencing and Genotyping, Culver City, CA). If sequences were pure and positively matched to *A. muciniphila* in GenBank by BLAST analysis, the nearly full-length 16S rRNA gene was sequenced with additional primers (515F [GTGCCAGCMGCCGCGGTAA], 806R [GGACTACHVGGGTWTCTAAT], and 8F or 1492R). Sequences associated with each isolate were then assembled in Geneious 7.1.3 and imported into ARB (67). In ARB, sequences were manually aligned with secondary structure constraints against the 16S rRNA gene sequence of *A. muciniphila* Muc^T^. To determine phylogroup affiliation based on 16S rRNA gene sequences, each isolate was added to our in-house database of *Akkermansia* 16S rRNA gene sequences as previously described (10, 15). Masked alignments were exported from ARB and imported into Kumar and colleagues’ (68) MEGA7, where phylogenetic reconstruction was performed using the maximum likelihood approach.

### Genome sequencing, assembly, annotation, and phylogenomics.

Eleven *Akkermansia* isolates were selected for genome sequencing across three different sequencing efforts. DNAs from strains CSUN-7 and CSUN-12 were sequenced according to the Illumina sequencing protocol described previously by Oliver and colleagues (69). To obtain enough DNA for this sequencing protocol, four 5-mL cultures grown overnight in BMM were extracted as described above, and extracts were pooled and concentrated using ethanol precipitation with 3 M sodium acetate. Illumina sequencing libraries were then prepared as described previously by Oliver and colleagues. DNA from strains CSUN-17, CSUN-19, CSUN-33, and CSUN-34 were sequenced according to methods described previously by Parker and colleagues (70). For both this and the following sequencing efforts, enough quality genomic DNA was obtained from a single 5-mL culture of each isolate grown in BMM and extracted as described above. The DNAs from the remaining isolates (CSUN-37, CSUN-50, CSUN-56, CSUN-58, and CSUN-59) were sequenced on an Illumina NextSeq 550 platform (2 by 150 bp) by the Microbial Genome Sequencing Center (Pittsburgh, PA, USA).

For assembly and annotation, paired fastq files for each isolate were submitted to PATRIC (v 3.6.3) (71) for their comprehensive genome analysis workflow that uses Unicycler (72) to assemble genomes and RASTtk (73) for annotation. For comparison, the nucleotide sequence file for *A. muciniphila* Muc^T^ ATCC BAA-835 (BioSample accession number SAMN00138213) was downloaded from GenBank and annotated identically to the novel isolate genomes, also using tools in PATRIC. To investigate the carbohydrate-degrading potential of each *Akkermansia* phylogroup, the assembled contigs of the new isolates (*n* = 11) were combined with 74 publicly available *Akkermansia* genomes (10, 15, 74) and submitted to the dbCAN meta server for CAZyme annotation (27–29). dbCAN uses three tools, HMMER (75), DIAMOND (76), and Hotpep (77), for automated carbohydrate-active enzyme (CAZyme) annotation. Annotations were considered only if they matched with at least two of the three tools. Individual count files were tabulated and compiled using a custom python script to generate a frequency table for all genomes (*n* = 85). The resulting table was sorted and trimmed to include only glycoside hydrolase (GH) annotations, and a heat map was constructed in R (78) using the heatmap.2 function in the gplots library (79). Cluster dendrograms in the heat map were calculated using average linkage hierarchical clustering based on Bray-Curtis dissimilarity matrices calculated using the vegan package, also in R (80). To determine if there were differences in the numbers of GH predictions between phylogroups, a Kruskal-Wallis test (kruskal.test) followed by Dunn’s test (dunn.test, method=‘bonferroni’) were performed in R.

For phylogenomic analysis, amino acid sequences of 49 ribosomal protein-coding genes (81) were extracted and concatenated from assembled genomes using the phylogenomics workflow in anvi’o (82). The concatenated fasta file was then imported into MEGA7 (68) and aligned using MUSCLE (83), and a phylogenetic tree was made using the maximum likelihood method (84) with 100 bootstraps.

### HMO growth experiments.

To determine if *Akkermansia* strains could grow using HMOs, we performed a series of growth experiments in a customized medium prepared by increasing the concentrations of threonine and tryptone (TT) in BMM (9). This medium, referred to here as BMM-TT (Table S1), was supplemented with individual HMOs before inoculation with the chosen *Akkermansia* strains. Five HMOs were tested, namely, 2′-FL, 3-FL, LNT, LNnT, and 6′-SL (Glycom, Hørsholm, Denmark). Lactose was also included in these growth experiments since it is the backbone of HMOs. Initially, one representative isolate of each phylogroup (AmI, *A. muciniphila* Muc^T^; AmII, *Akkermansia* sp. strain CSUN-17; AmIII, *Akkermansia* CSUN-56; AmIV, *Akkermansia* CSUN-19) (Table S2) was grown overnight (18 to 24 h) in BMM at 37°C under an atmosphere of N_2_-CO_2_ (70:30, vol/vol). Cultures were then standardized to an OD_600_ of 0.5 in fresh BMM and used to inoculate (10%) 200 μL of BMM-TT or BMM-TT supplemented with 20 mM each HMO (or lactose) in 96-well microtiter plates (Falcon; Corning Incorporated, Corning, NY, USA) in triplicate. Wells were overlaid with 30 μL of filter-sterilized mineral oil to prevent evaporation over the 48-h incubation period. After 48 h of anaerobic (N_2_-CO_2_-H_2_ [80:15:5, vol/vol]) incubation at 37°C in a Bactron IV anaerobic chamber (Sheldon Manufacturing Inc., Cornelius, OR), plates were shaken for 10 s, and the OD_600_ was determined using a Spectramax microplate reader (Molecular Devices, San Jose, CA, USA). Growth was determined as the ΔOD_600_, i.e., the change in the OD_600_ of growth in BMM-TT supplemented with the HMOs relative to the growth in HMO-unsupplemented BMM-TT (i.e., BMM-TT + HMO OD_600_ − BMM-TT OD_600_). If the OD_600_ was >1.0, samples were diluted in half with fresh medium and reread. Each experiment was conducted in triplicate and repeated at least two times. To test for differences in growth across strains, we used repeated-measures analysis of variance (ANOVA) followed by Tukey’s honestly significant difference (HSD) test as appropriate. Uninoculated controls were included in each experiment and remained negative for growth.

To verify the degradation of three HMOs (2′-FL, LNT, and 6′-SL), the above-described experiments were repeated in 1.5 mL of BMM-TT supplemented with 4 mM HMO. These experiments were conducted in 24-well microtiter plates (Costar; Corning Incorporated, Corning, NY, USA) sealed with Microseal A film (Bio-Rad Laboratories Inc., Hercules, CA, USA) instead of mineral oil. Plates were incubated, and the OD_600_ and ΔOD_600_ were measured after 48 h as described above. For glycoanalytics, 0.5-mL aliquots were taken at time zero and 48 h after incubation, transferred to Eppendorf tubes, and centrifuged at 10,000 × *g* for 3 min at 4°C. The cell-free supernatants were stored at −20°C for glycoanalytics as described below. To compare growth, statistical analysis was conducted as described above.

### HMO quantification.

Culture supernatants were collected at time zero and after 48 h of incubation to measure the degradation of 2′-FL, LNT, and 6′-SL. In addition to each parent HMO, individual sugars (with the exception of GlcNAc from LNT) of the three HMOs were also quantitatively measured using high-performance anion-exchange chromatography with pulsed amperometric detection (HPAEC-PAD) (85, 86). Frozen, cell-free spent culture media were thawed in a water bath, vortexed thoroughly to make a uniform mixture, and centrifuged at 7,000 × *g* for 5 min at 10°C, and 1 μl of the spent culture medium was injected into the HPAEC-PAD instrument for the detection of the above-mentioned sugars.

Carbohydrate analysis was done on a Dionex-ICS3000 system (Thermo Scientific, Sunnyvale, CA, USA) using a CarboPac PA-1 column (4 mm by 250 mm) attached to a Carbo PA1-guard column (4 mm by 50 mm). The detection of monosaccharides and oligosaccharides was done using standard Quad potential for carbohydrate analysis as supplied by the manufacturer. A gradient mixture of two solvents along with high-performance liquid chromatography (HPLC)-grade water was used for the optimum separation of monosaccharides and oligosaccharides present in the sample. Solvent A (water), solvent B (100 mM NaOH plus 7 mM sodium acetate [NaOAc]), and solvent C (100 mM NaOH plus 250 mM NaOAc) were used as elution solvents at a flow rate of 1.0 mL/min. Gradient mixture details are given in Table S4 in the supplemental material. Sugars were quantified by comparison with the area under the peaks from a standard mixture of fucose, galactose, glucose, 3-FL, lactose, 2′-FL, LNnT, LNT, sialic acid (Neu5Ac), 6′-SL, and 3-SL. Representative chromatograms are presented in Fig. S1. To determine the percentage of HMOs utilized, the amount remaining after 48 h of incubation was divided by the amount at time zero and multiplied by 100 [(HMO 48 h/HMO 0 h) × 100].

### Data availability.

The data that support the findings of this study are openly available in the NCBI BioProject database under accession number PRJNA609771.
