# Supplementary material for: Utilization Efficiency of Human Milk Oligosaccharides by Human-Associated Akkermansia Is Strain Dependent
Source: Appl Environ Microbiol. 2022 Jan 11;88(1):e01487-21. doi: 10.1128/AEM.01487-21 (PMC8752153; doi:10.1128/AEM.01487-21)
Supplement: Supplemental file 1 — Tables S1 to S4, Fig. S1 and S2. Download AEM.01487-21-s0002.pdf, PDF file, 0.6 MB [file aem.01487-21-s0002.pdf]

Supplementary material

Utilization efficiency of human milk oligosaccharides by human-associated *Akkermansia* is strain-dependent

Estefani Luna[1#], Shanthi G. Parkar[1#], Nina Kirmiz[1], Stephanie Hartel[1], Erik Hearn[1], Marziiah Hossine[1], Arinnae Kurdian[1], Claudia Mendoza[1], Katherine Orr[1], Loren Padilla[1], Katherine Ramirez[1], Priscilla Salcedo[1], Erik Serrano[1], Biswa Choudhury[2], Mousumi Paulchakrabarti[2], Steven Huynh[3], Craig T. Parker[3], Kerry Cooper[4], and Gilberto E. Flores[1]*

[1]*Department of Biology, California State University, Northridge, Northridge, CA 91330-8303.*

[2]*GlycoAnalytics Core, UC San Diego, Health Sciences, La Jolla, CA 92093-0687.*

[3]*Produce Safety and Microbiology Research Unit, Western Regional Research Center, Agricultural Research Service, US Department of Agriculture, Albany, CA 94710.*

[4]*School of Animal and Comparative Biomedical Sciences, University of Arizona, Tucson, AZ 85721.*

[*]Corresponding author: gilberto.flores@csun.edu (818) 677-4276

[#]Authors contributed equally

Table S1. Components of culture media used in the *Akkermansia* growth studies.

**Component**	BMM (amount/L)	BMM-TT (amount/L)
KH_2PO_4	0.4 g	0.4 g
Na_2HPO_4	0.53 g	0.53 g
NH_4Cl	0.3 g	0.3 g
NaCl	0.3 g	0.3 g
$MgCl_2 \cdot 6H_2O$	0.1 g	0.1 g
$NaHCO_3$	0.4 g	0.4 g
Resazurin	0.001 g	0.001 g
Trace mineral solution[1]	10 mL	10 mL
L-threonine	1 mM	11 mM
Tryptone	10 g	18 g
$Na_2S \cdot 9H_2O$[1]	0.5 g	0.5 g
Purified mucin[1]	5 g	5 g
Noble agar[2]	1.2 g	1.2 g

Trace mineral solution prepared using previously described proportions [1].

Purified mucin was prepared from hog gastric mucin (type III; Sigma-Aldrich, St. Louis, MO) as described previously [10].

[1] Added from sterile stock after autoclaving

[2] Added only to the solid media

BMM = Basal mucin medium

BMM-TT = Basal mucin medium- threonine and tryptone

Table S2. Seventeen *Akkermansia* strains were isolated from healthy adult humans of various sex, age, diet, and ethnicity. At least one representative of each phylogroup was isolated and had their genomes sequenced (*).

Isolate (phylogroup)	Subject Sex	Subject Age	Subject Diet	Subject Ethnicity	16S rRNA gene accession #	BioSample #
Akkermansia CSUN-7* (AmI)	Male	63	Omnivore	Caucasian	MK577303	SAMN14614183
Akkermansia CSUN-12* (AmI)	Male	22	Omnivore	Hispanic	MK577304	SAMN14614184
Akkermansia CSUN-17* (AmII)	Male	32	Omnivore	Hispanic	MK577312	SAMN14614185
Akkermansia CSUN-19* (AmIV)	Male	65	Omnivore	Caucasian	MT274551	SAMN14614186
Akkermansia CSUN-23 (AmI)	Male	27	Omnivore	Caucasian	MK577305	NA
Akkermansia CSUN-27 (AmI)	Male	28	Omnivore	Caucasian	MK577306	NA
Akkermansia CSUN-28 (AmI)	Male	33	Omnivore	Hispanic	MK577309	NA
Akkermansia CSUN-31 (AmI)	Female	23	Vegan	Hispanic	MK577310	NA
Akkermansia CSUN-33* (AmI)	Male	22	Vegetarian	Hispanic	MK577311	SAMN14614187
Akkermansia CSUN-34* (AmII)	Male	22	Omnivore	Hispanic	MK577308	SAMN14614188
Akkermansia CSUN-36 (AmI)	Female	39	Omnivore	Caucasian	MK577307	NA
Akkermansia CSUN-37* (AmIV)	Male	Unknown	Omnivore	Unknown	MT274548	SAMN14614189
Akkermansia CSUN-50* (AmII)	Female	23	Omnivore	Hispanic	MT274549	SAMN14614190
Akkermansia CSUN-54 (AmI)	Female	48	Omnivore	Hispanic	MT274552	NA

Akkermansia CSUN-56* (AmIII)	Female	21	Omnivore	Caucasian	MT274553	SAMN14614191
Akkermansia CSUN-58* (AmII)	Female	33	Omnivore	Hispanic	MT274550	SAMN14614192
Akkermansia CSUN-59* (AmI)	Female	59	Omnivore	Caucasian	MT274547	SAMN14614193

*denotes isolates with draft genome sequences

Table S3. Gradient used in the HPAEC-PAD analysis of culture supernatant for quantification of monosaccharides and oligosaccharides. Solvent A was HPLC water, Solvent-B was 100mM NaOH + 7mM NaOAc, and Solvent-C was 100mM NaOH + 250mM NaOAc.

Time (min)	Solvent-A	Solvent-B	Solvent-C
0	80%	19%	1%
20.0	71%	19%	10%
60.0	0%	19%	81%
62.0	80%	19%	1%
77.0	80%	19%	1%

a

**Sample Name:**	HMO-0.5ug+Mono-0.1ug-New		**Sample No.:**	6
**Sequence Name:**	060520-Mono-test			
**Program Method:**	HMO-77min-method-032620		**Injection vol.:**	100.0
**Quantitation Method:**	monos		**Dilution Factor:**	1.0000
**Date Time Collected:**	6/5/2020	3:27 PM	**Sample Wt.:**	1.0000
**System Operator:**	UNIVERSITY OF CALIFO		**Sample Amt.:**	1.0000

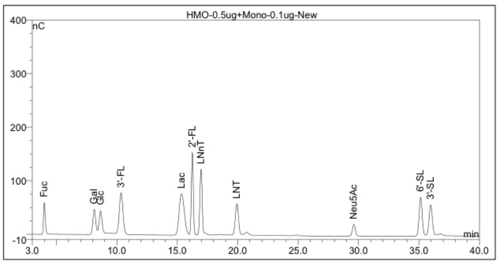

Peak No.	Name	Retention Time min	Area n.a.	Rel.Area %	Height nC	Amount ug
1	Fuc	3.99	10.053	4.49	59.193	0.1000
2	Gal	8.13	12.821	5.72	46.832	0.1000
3	Glc	8.65	13.480	6.01	44.343	0.1000
4	3'-FL	10.35	28.080	12.53	77.787	0.5000
5	Lac	15.35	38.096	17.00	76.834	0.5000
6	2'-FL	16.24	29.230	13.04	153.740	0.5000
7	LNnT	16.97	27.660	12.34	122.647	0.5000
8	LNT	19.93	17.886	7.98	58.223	0.5000
9	Neu5Ac	29.61	6.447	2.88	22.007	0.1000
10	6'-SL	35.13	21.660	9.66	72.103	0.5000
11	3'-SL	35.97	18.717	8.35	57.901	0.5000

**Sample Name:**	dB-2b-1uL		**Sample No.:**	7
**Sequence Name:**	060520-Mono-test			
**Program Method:**	HMO-77min-method-032620		**Injection vol.:**	100.0
**Quantitation Method:**	monos		**Dilution Factor:**	1.0000
**Date Time Collected:**	6/5/2020	4:48 PM	**Sample Wt.:**	1.0000
**System Operator:**	UNIVERSITY OF CALIFO		**Sample Amt.:**	1.0000

b

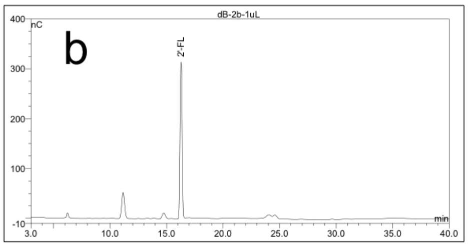

Peak No.	Name	Retention Time min	Area n.a.	Rel.Area %	Height nC	Amount ug
1	2'-FL	16.28	68.955	100.00	313.948	1.1795

**Sample Name:**	dB-2c-1uL		**Sample No.:**	8
**Sequence Name:**	060520-Mono-test			
**Program Method:**	HMO-77min-method-032620		**Injection vol.:**	100.0
**Quantitation Method:**	monos		**Dilution Factor:**	1.0000
**Date Time Collected:**	6/5/2020	6:08 PM	**Sample Wt.:**	1.0000
**System Operator:**	UNIVERSITY OF CALIFO		**Sample Amt.:**	1.0000

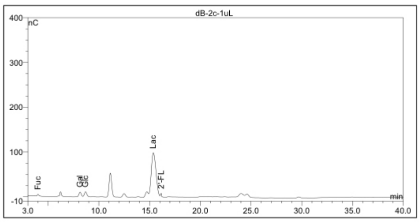

Peak No.	Name	Retention Time min	Area n.a.	Rel.Area %	Height nC	Amount ug
1	Fuc	4.00	0.587	0.98	3.716	0.0058
2	Gal	8.14	2.695	4.49	10.215	0.0210
3	Glc	8.68	3.031	5.04	10.888	0.0225
4	Lac	15.37	52.853	87.96	98.563	0.6937
5	2'-FL	16.15	0.924	1.54	7.954	0.0158

**Sample Name:**	dC-6a-1uL		**Sample No.:**	12
**Sequence Name:**	060920-Mono-test			
**Program Method:**	HMO-77min-method-032620		**Injection vol.:**	100.0
**Quantitation Method:**	monos		**Dilution Factor:**	1.0000
**Date Time Collected:**	6/10/2020	2:09 AM	**Sample Wt.:**	1.0000
**System Operator:**	UNIVERSITY OF CALIFO		**Sample Amt.:**	1.0000

c

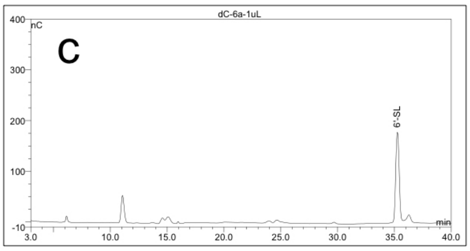

Peak No.	Name	Retention Time min	Area n.a.	Rel.Area %	Height nC	Amount ug
1	6'-SL	35.27	62.729	100.00	177.781	1.4830

**Sample Name:**	dC-6d-1uL		**Sample No.:**	14
**Sequence Name:**	060920-Mono-test			
**Program Method:**	HMO-77min-method-032620		**Injection vol.:**	100.0
**Quantitation Method:**	monos		**Dilution Factor:**	1.0000
**Date Time Collected:**	6/10/2020	4:50 AM	**Sample Wt.:**	1.0000
**System Operator:**	UNIVERSITY OF CALIFO		**Sample Amt.:**	1.0000

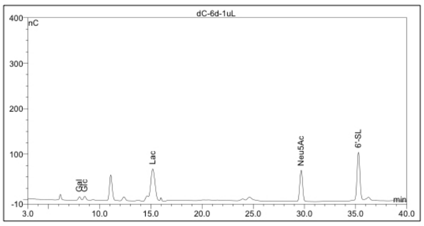

Peak No.	Name	Retention Time min	Area n.a.	Rel.Area %	Height nC	Amount ug
1	Gal	8.03	1.998	2.14	7.697	0.0177
2	Glc	8.54	2.258	2.42	8.316	0.0198
3	Lac	15.18	34.151	36.56	70.541	0.4653
4	Neu5Ac	29.68	21.448	22.96	68.086	0.3635
5	6'-SL	35.28	33.563	35.93	105.134	0.7934

**Sample Name:**	dC-La-1uL		**Sample No.:**	7
**Sequence Name:**	060920-Mono-test			
**Program Method:**	HMO-77min-method-032620		**Injection vol.:**	100.0
**Quantitation Method:**	monos		**Dilution Factor:**	1.0000
**Date Time Collected:**	6/9/2020	7:27 PM	**Sample Wt.:**	1.0000
**System Operator:**	UNIVERSITY OF CALIFO		**Sample Amt.:**	1.0000

d

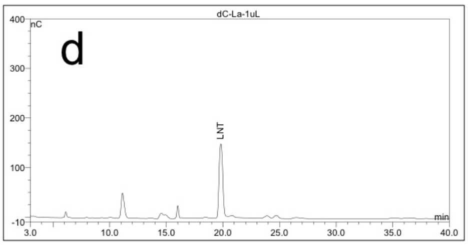

Peak No.	Name	Retention Time min	Area n.a.	Rel.Area %	Height nC	Amount ug
1	LNT	19.81	55.528	100.00	149.081	1.6002

**Sample Name:**	dC-Le-1uL		**Sample No.:**	11
**Sequence Name:**	060920-Mono-test			
**Program Method:**	HMO-77min-method-032620		**Injection vol.:**	100.0
**Quantitation Method:**	monos		**Dilution Factor:**	1.0000
**Date Time Collected:**	6/10/2020	12:49 AM	**Sample Wt.:**	1.0000
**System Operator:**	UNIVERSITY OF CALIFO		**Sample Amt.:**	1.0000

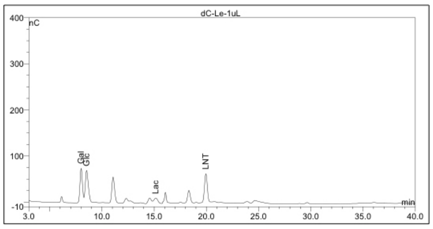

Peak No.	Name	Retention Time min	Area n.a.	Rel.Area %	Height nC	Amount ug
1	Gal	8.01	20.307	29.37	74.682	0.1795
2	Glc	8.53	24.134	34.91	70.017	0.2112
3	Lac	15.16	5.206	7.53	11.563	0.0709
4	LNT	19.95	19.484	28.18	62.257	0.5615

Supplementary Figure S1. Representative chromatograms from a standard mix of human milk oligosaccharides (A), and the zero time and 48-h cultures of mucin-containing media supplemented with 2’-fucosyllactose (B), 6’-sialyllactose (C) and lacto-N-tetraose (D).

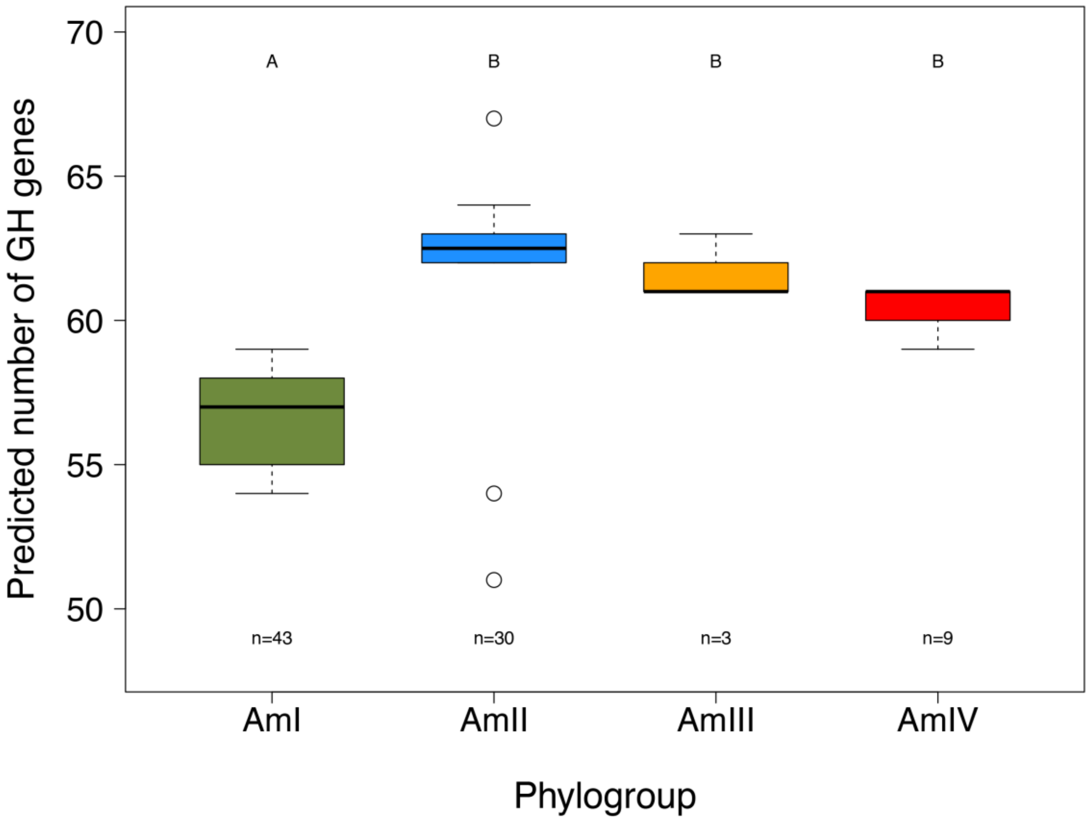

Supplemental Figure 2. *Akkermansia* phylogroup AmI possesses the least number of glycoside hydrolase (GH) gene annotations compared to the other phylogroups. Letters above each box indicate results of the pairwise Dunn’s Test; boxes with different letters indicate significant differences following correction of P-values using the Bonferroni correction ($P_{corrected} < 0.01$).
